# Usability testing a web application to support evidence-based commissioning decisions for implementing mobile stroke units

**DOI:** 10.1038/s41746-025-01691-2

**Published:** 2025-05-09

**Authors:** Lisa Moseley, Anna Laws, Michael Allen, Gary A. Ford, Martin James, Stephen McCarthy, Graham McClelland, Laura J. Park, Kerry Pearn, Daniel Phillips, Christopher Price, Lisa Shaw, Phil White, David Wilson, Peter McMeekin, Jason Scott

**Affiliations:** 1 Faculty of Health and Life Sciences, Northumbria University, Newcastle upon Tyne, UK; 2 University of Exeter Medical School, Exeter, UK and NIHR South West Peninsula Applied Research Collaboration (ARC), Exeter, UK; 3 Oxford University Hospitals NHS Foundation Trust, Oxford, UK; 4 Radcliffe Department of Medicine, University of Oxford, Oxford, UK; 5 Royal Devon and Exeter Hospital, Exeter, UK; 6 East of England Ambulance Service NHS Trust, Cambridgeshire, UK; 7 Stroke Research Group, Population Health Sciences Institute / Translational and Clinical Research Institute, Newcastle University, Newcastle upon Tyne, UK; 8 Stroke Service User Voice Group, Newcastle upon Tyne, UK

**Keywords:** Health policy, Health services, Stroke

## Abstract

Commissioning of innovations in healthcare is a complex socio-technical process, ideally informed by high quality evidence. However, evidence is not always prepared and presented in a format usable for commissioning decisions. Agile methodology, combined with qualitative co-design, were used to develop a digital web application incorporating machine learning models of stroke outcomes to inform commissioning decisions for the implementation of mobile stroke units (MSUs) in England, followed by usability testing using think aloud methodology. Sixteen stakeholders involved in developing consensus on model parameters and pathways participated with data thematically analysed. Required improvements to the web application were identified and novel insights into the complexity of context-specific commissioning decisions were generated, which also informed participants’ views on the viability of MSUs. This study provides empirical evidence in support of developing innovative and accessible digital dissemination methods to engage with commissioning processes and prospectively understand commissioning challenges.

## Introduction

Stroke is one of the leading causes of death and disability worldwide^1^. Strokes are caused either by a clot, or a bleed, in the brain and are characterised by rapid onset of symptoms, which vary in severity and can impact all areas of function including mobility and speech^2^. Long-term effects following a stroke can be severe, requiring ongoing health treatment as well as social care support^3^. It is estimated that the number of strokes in the United Kingdom (UK) will rise by 59% by 2035, from the 2020 level of approximately 100,000 a year^4^. This makes stroke an important health concern due to the individual impact, alongside the economic burden. Treatment for ischaemic strokes, where the stroke is caused by a clot, can reduce, and even fully diminish, the impacts of a stroke. However, this treatment is time sensitive, making rapid diagnosis and intervention imperative^5^.

Mobile stroke units (MSUs) have been growing in popularity as a means to diagnose and begin treatment of ischaemic strokes in pre-hospital settings, leading to the European Stroke Organisation (ESO) to recommend consideration of MSU deployment^6^. MSUs are specialist ambulances that have a computed tomography (CT) or CT angiography scanner, capable of scanning the head with the latter using contrast to visualise blood vessels, and MSUs also have stroke specialist staffing and point of care testing^7–14^. MSUs have focused on the treatment of ischaemic stroke, and trials have demonstrated that MSUs can speed up to time to treatment with intravenous thrombolysis (IVT), a medication used in some cases to dissolve clots^9–13^. Another treatment, mechanical thrombectomy (MT), is used for some patients where the clot is occluding a large intracranial vessel, known as a large vessel occlusive (LVO) stroke. MT is performed by hospital-based neurointerventionalists who manually retrieve the clot in a specialist facility to restore blood flow^15^. In the UK, like most countries, MT is only available at certain specialist centres, comprehensive stroke centres (CSC), usually based in urban areas. However, patients with suspected stroke are currently often transported by the ambulance service to their nearest hospital with standard stroke services available, which can be an acute stroke unit (ASU) without MT capabilities and thus requires an additional ambulance transfer of patients with LVO stroke to CSCs. While initial trials of MSUs focused on the increased speed in which IVT was given, more recent evidence has found there is also the potential for MSUs to speed up access to MT, although that has not been consistently evidenced^7,8,14^. Despite the evidence favouring MSU in certain settings and healthcare systems and ESO guidance, they are currently not commissioned in the NHS.

Commissioning has been a part of the National Health Service (NHS) since its inception in 1948, with services being provided by local authorities, hospitals and independent practitioners (i.e., general practitioners), each having to submit estimated costs, but largely managing their own budgets locally^16^. In the 1990s, the NHS was changed to a quasi-market to encourage competition between providers, aiming to reduce costs and improve efficiencies^17^. Arguments for the quasi-marketisation championed the purchasing of services being separated from those providing the service, with the view that this would improve equitable access to services universally, without influence from those providing services, particularly clinicians^18^.

Commissioning of services within the NHS remains complex, with decisions being made centrally and by local commissioners^19^. Increasingly, there is also tension between cost-effectiveness commissioning compared with values-based commissioning, which differ in emphasis; the former focuses on societal benefits and the latter is intended to consider individual patient perspectives^20^. Evidence-based policy is key to all service planning within the NHS and is a key consideration in commissioning decisions^21^. The potential benefits of evidence-based commissioning decisions include a greater chance of a service being successfully implemented as well as more efficient use of public funds within the health service^22,23^. Despite the benefits of using data in commissioning decisions the reality of how commissioning decisions are made is much more complex^24,25^. Instead of being a transactional approach, commissioning involves close relational interaction between commissioners, providers of services and often patients and the public^26,27^.

Numerous barriers exist to commissioners using evidence in their decision-making, including research articles being behind paywalls, research being clinically focused rather than commissioner focused, lack of localised knowledge or context in research, and the effort required to understand academic research^28^. A number of facilitators to data-informed commissioning have been identified including making data presented manipulatable, showing the data meaning at local levels, and making the data easy to interpret^29^. Alongside these complexities in the commissioning of services there is now an emphasis on the co-production of health and care services, requiring services to be informed by relevant stakeholders^30^. However the use of co-production has posed further challenges to commissioners with difficulties involving stakeholders in an already complex commissioning process, the work required for co-production being unfeasible within commissioner time constraints, and the inflexibility of the commissioning processes to respond to additional input(s)^25,31^.

To address these challenges, researchers are increasingly required to develop new approaches to sharing evidence created through research that can directly inform commissioning decisions, such as more accessible data summaries^32^. Web applications are one such approach and are increasingly being used to provide end-user access to complex models (e.g., see Narayanan et al.^33^) and have key advantages for making models available. These advantages include reduced barriers to end-user engagement, no specialist software or hardware needs for the end-user, rapid prototyping and development, the ability to work across multiple platforms (e.g., PC, tablet and phone), centralised updates/maintenance, immediate deployment of fixes or enhanced functionality, and scalable infrastructure which can adapt to demand. Within healthcare informatics, there is now an increased emphasis on sharing the underlying code of models^34^.

This paper describes the approach taken to developing a web application for modelling the effectiveness of MSUs in England and examines the usability of the web application for informing commissioning decisions.

## Results

**Fig. 1:**
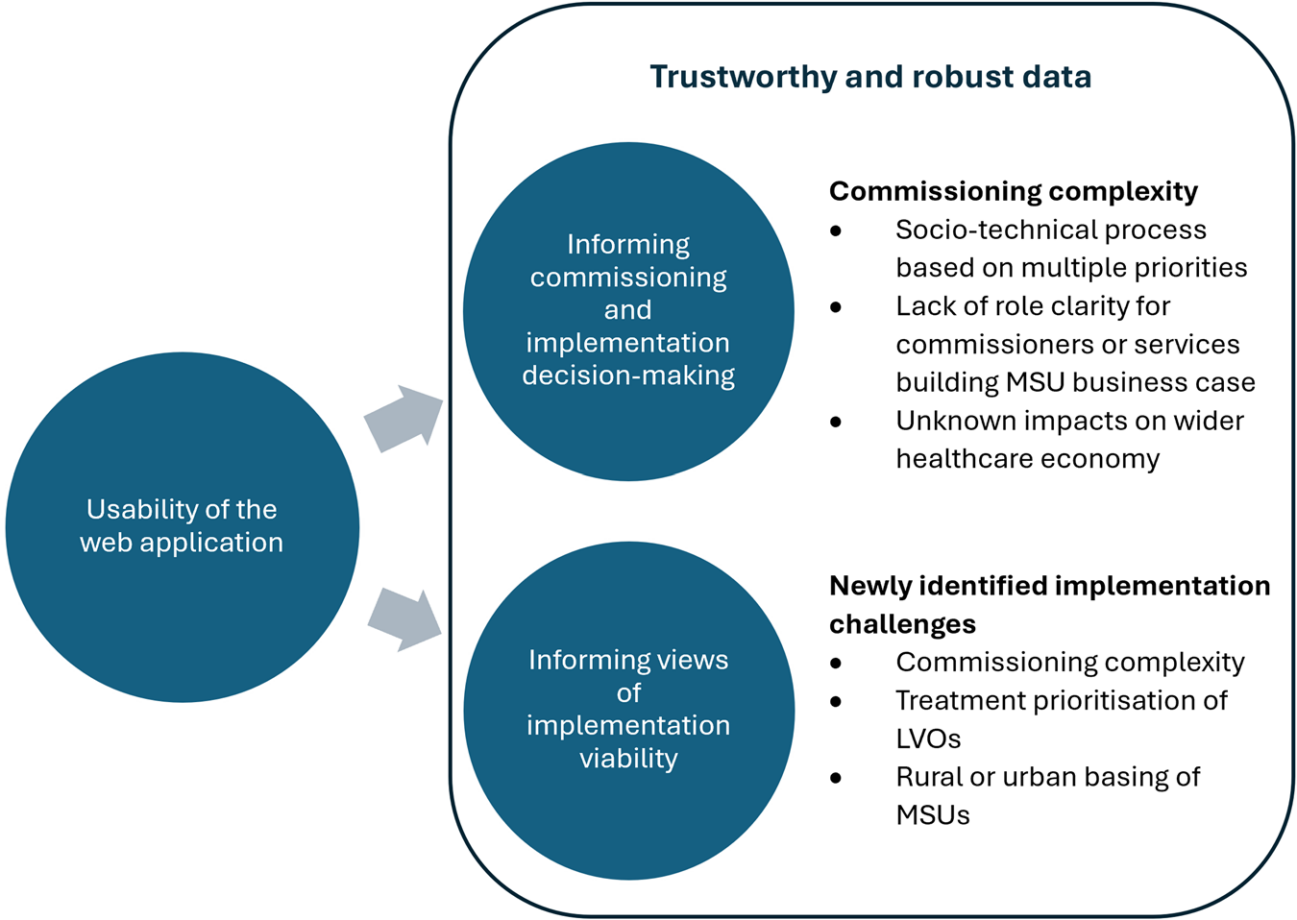
Thematic structure of the findings. MSU Mobile stroke unit, LVO Large vessel occlusion.

Sixteen people participated in usability testing, consisting of patient and public involvement representatives (PPI) (*n* = 6, 38%), stroke or critical care physicians (*n* = 6, 38%), and ambulance service staff with front-line clinical and senior management expertise (*n* = 4, 25%). Interviews lasted between 38 and 89 min (mean = 58 min). Fig. 1 presents an overview of the thematic structure of the findings.

We identified where users were able to successfully navigate and operate the web application, as well as areas that its usability could be improved. Together, these form the first theme: *Usability of the web application*. Central to this theme were the importance of clear user instructions and participants’ preferences for data presentation. The second theme developed, *Informing commissioning and implementation decision-making*, examines how usability testing of the web application provided insight to the complexity of commissioning and implementation of MSUs, and allowed for a deeper exploration of how various stakeholders considered how the web application could be used. Related to this was a third theme, *Informing views on intervention viability*, that captures how usability testing allowed a further examination of whether MSUs could or should be commissioned. Finally, we developed a single cross-cutting theme that interacted with and was central to the second and third major themes; *Trustworthy and robust data*. This theme, particularly focusing on the need to include health economics data on cost-effectiveness of MSUs, reinforces the need for commissioning and implementation decisions to be data-driven and was seen as a requirement for participants to provide conclusive views on whether MSUs should be implemented. Images of the web application are included to contextualise findings. Outcomes shown in images are for illustrative purposes only and not necessarily representative of the final models.

### Usability of the web application

The overall view of the web application was positive with most participants quickly able to use it themselves after a brief guided tutorial by researchers. However, participants highlighted that should the web application be distributed widely it would not be feasible to give each individual a tutorial and as such would benefit from a recorded video tutorial, as well as written instructions with screenshots. This concern was discussed by a stroke consultant:

*“You are here, you explained it to me, and I found it very easy. So the question is, somebody who has not seen this and is seeing them for the first time, how do you make it user-friendly for them?…People differ, some people like that written instruction…with some images attached…Others would like the video…I think you need to cater for those two groups…” (Participant 13, stroke physician)*.

**Fig. 2:**
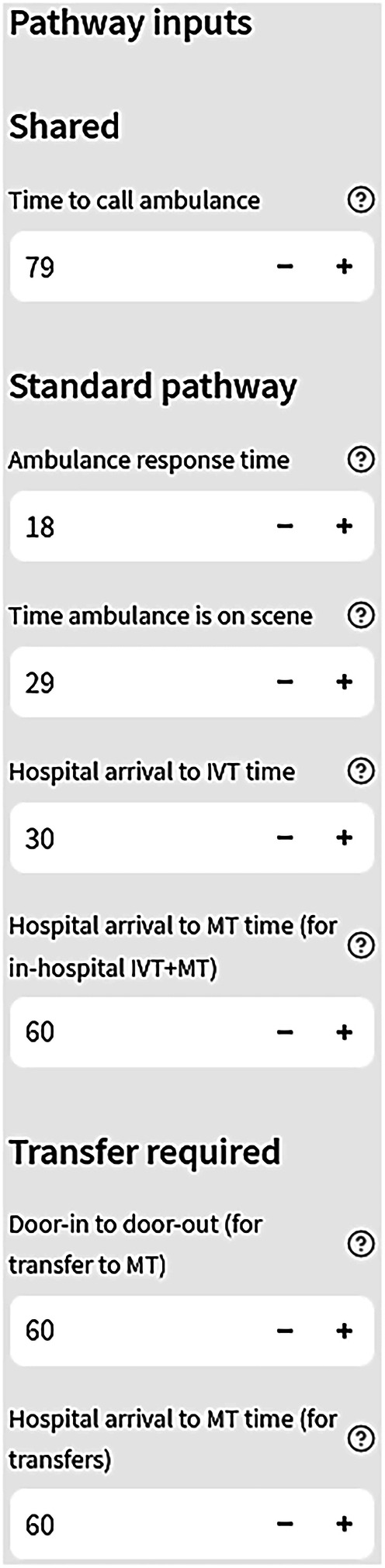
Process for modifying pathway parameters within the web application for the standard pathway. Hovering the cursor over the question marks repeated the parameter headings. Additional parameters were included for MSU inputs (not pictured here). Not all model parameters were modifiable, such as distance and travel times. Travel times between all 32,843 lower super output areas in England and hospitals were calculated using open street map data and Routino routing software, with travel times calibrated against Google Maps.

When using the web application, participants particularly found the selection of model parameters intuitive, where they were able to easily modify some time parameters used in the modelling (see Fig. 2). This allowed participants to tailor the models, for instance by amending the ambulance response time using publicly available data on response times to stroke calls within their region. Participants felt that the modifiable parameters needed a brief explanation of how the parameter interacted with the pathway and evidence-based justification for default values, such as whether previous stroke data had been used to obtain average values. These observations were explained by a stroke physician and an ambulance service staff member:

*“…where there’s this little question mark [above each parameter]…that’ll be nice if the question mark said, you know, the clarification of what it meant…” (participant 9, stroke or critical physician)*

*“Is that just a random figure or is that based on data? Is that currently where we think we are [in relation to ambulance response times]?” (Participant 2, ambulance service staff)*

**Fig. 3:**
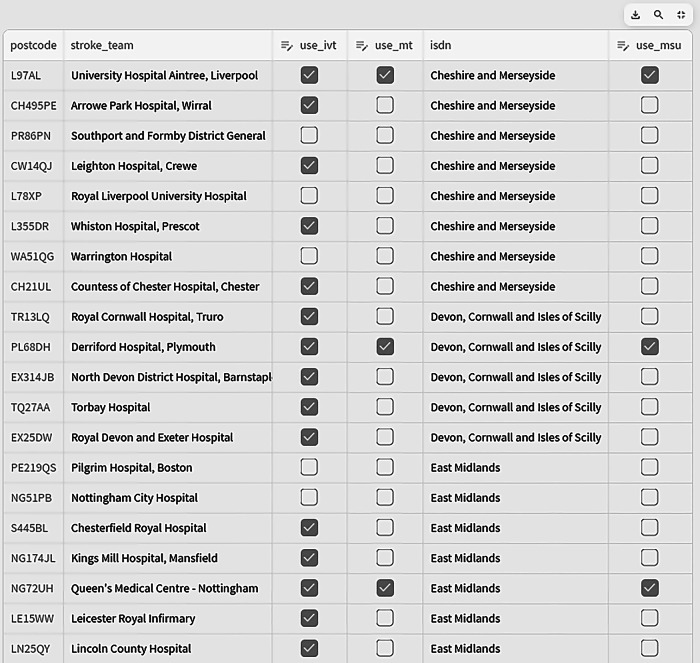
Presentation of potential locations for modelling MSU outcomes. Locations could be individually (de)selected by (un)ticking boxes in the right-hand side column.

Observing participant interactions with the web application revealed that they sometimes struggled to select the base location for an MSU, having to manually deselect default sites from a list of 128 locations. Despite being shown how to do this as part of the tutorial, some participants were unsure which boxes to (un)tick, and it was time-consuming for people to deselect multiple default options (Fig. 3).

**Fig. 4:**
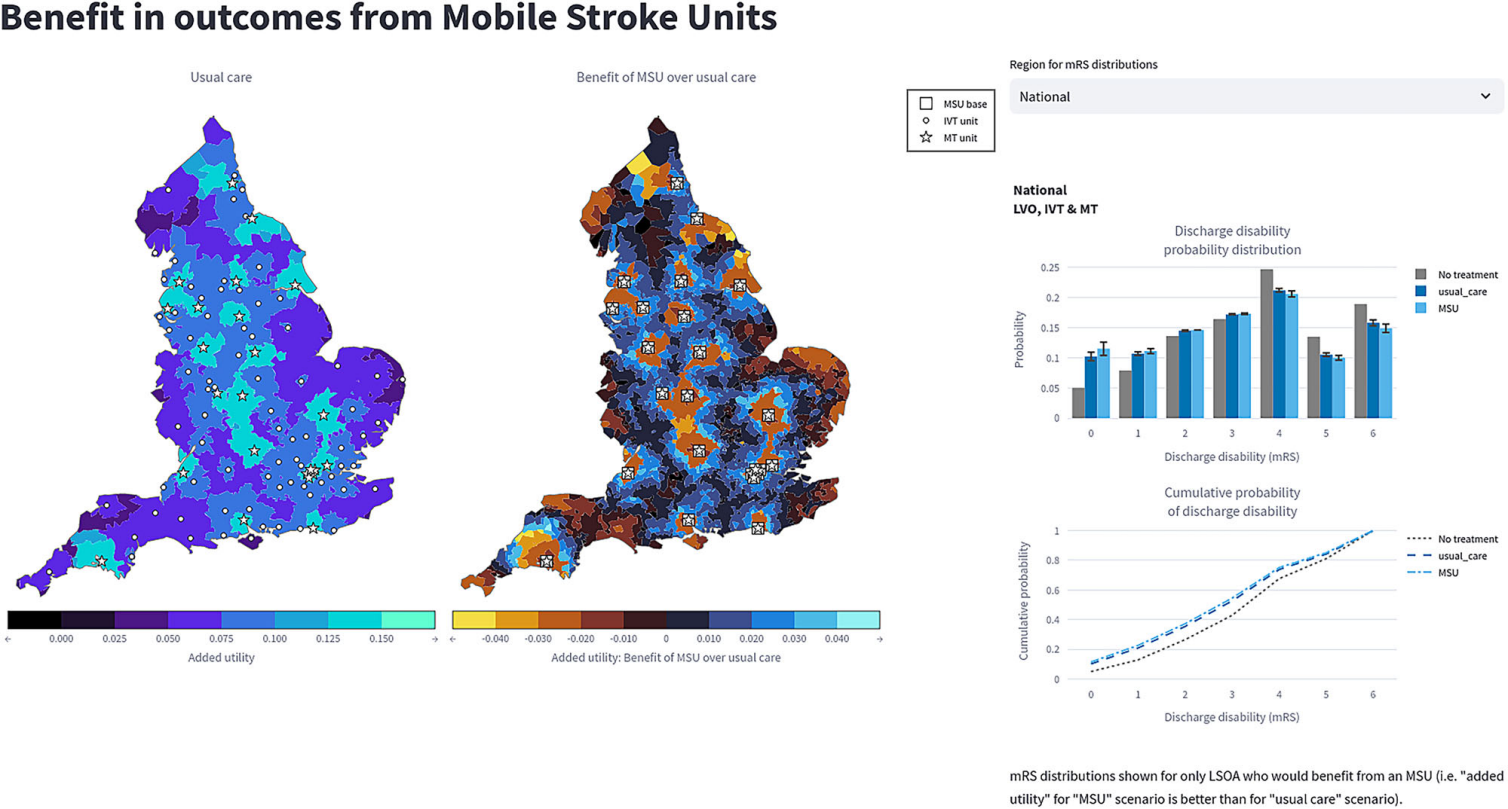
Web application interface. Web application Includes maps and graphs demonstrating outcomes associated with implementing MSUs. All maps include zoom functionality.

Within the web application, data were presented in multiple formats including maps, graphs and tables (Fig. 4). Observing participant interactions with the web application it was apparent that maps were the most accessible format with participants paying little attention to the graphs. Participants also did not interact with the data tables, consisting of 51 columns and representing all underlying data for the models, but they were deemed to be important (see *Trustworthy and rigorous data* theme).

“I did listen to what you described about the graphs on the right-hand side. But based on [the maps], you can predict what impact (MSUs) will have, I think. And that’s what [commissioners] need. So [commissioners] need something that’s visual. [Commissioners] need something that has some numbers associated with it, but also, because of the way that you’ve built this system, and I have to say, huge congratulations because it’s pretty intuitive, even with a very small amount of demonstration, you can get people to understand what their region looks like”. (Participant 14, stroke or critical care physician).

Whilst the maps received most attention, participants often struggled to understand the concept of utility shift—a change in quality of life measured through health utility scores—which was used as the default outcome comparison with usual care. Even with explanation of utility shift by the researchers, participants found it difficult to translate it into the actual benefit to patients when using the web application. While some stroke physicians were more familiar with the concept of utility shift this was not universal across all stroke physicians, restricting the usability of the web application to understand potential impact on patient outcomes. Concerns about utility shift and how understandable this would be were summarised by an ambulance service staff member:

*“I think…the biggest unanswered question as to what…the colours are lovely, and they’re dramatic and it makes you think, “Oh, wow, that’s a big difference.” But what is the difference?…I think I get my head around [utility shift]. I still think that’s- as someone lay, or whoever, or not even necessarily lay, that’s going to be their main question” (Participant 4, ambulance service staff)*.

Despite the concerns regarding the understanding of utility shift, as represented by colours on a map, participants were very positive about the use of the coloured map to visually represent the data quickly as well as being more thought provoking as explained by a stroke physician, *“it’s a really nice way of showing it…It makes it visibly more thought-provoking” (Participant 8, stroke or critical care physician)*.

Participants were also able to provide a number of helpful suggestions about changes to the maps that could enhance the usability of the web app. Suggestions included mapping on major road systems which would help users orientate more quickly as well as consider travel infrastructure in the area and including the locations of ASU on the MSU map, as this was currently only shown on the usual care map.

**Fig. 5:**
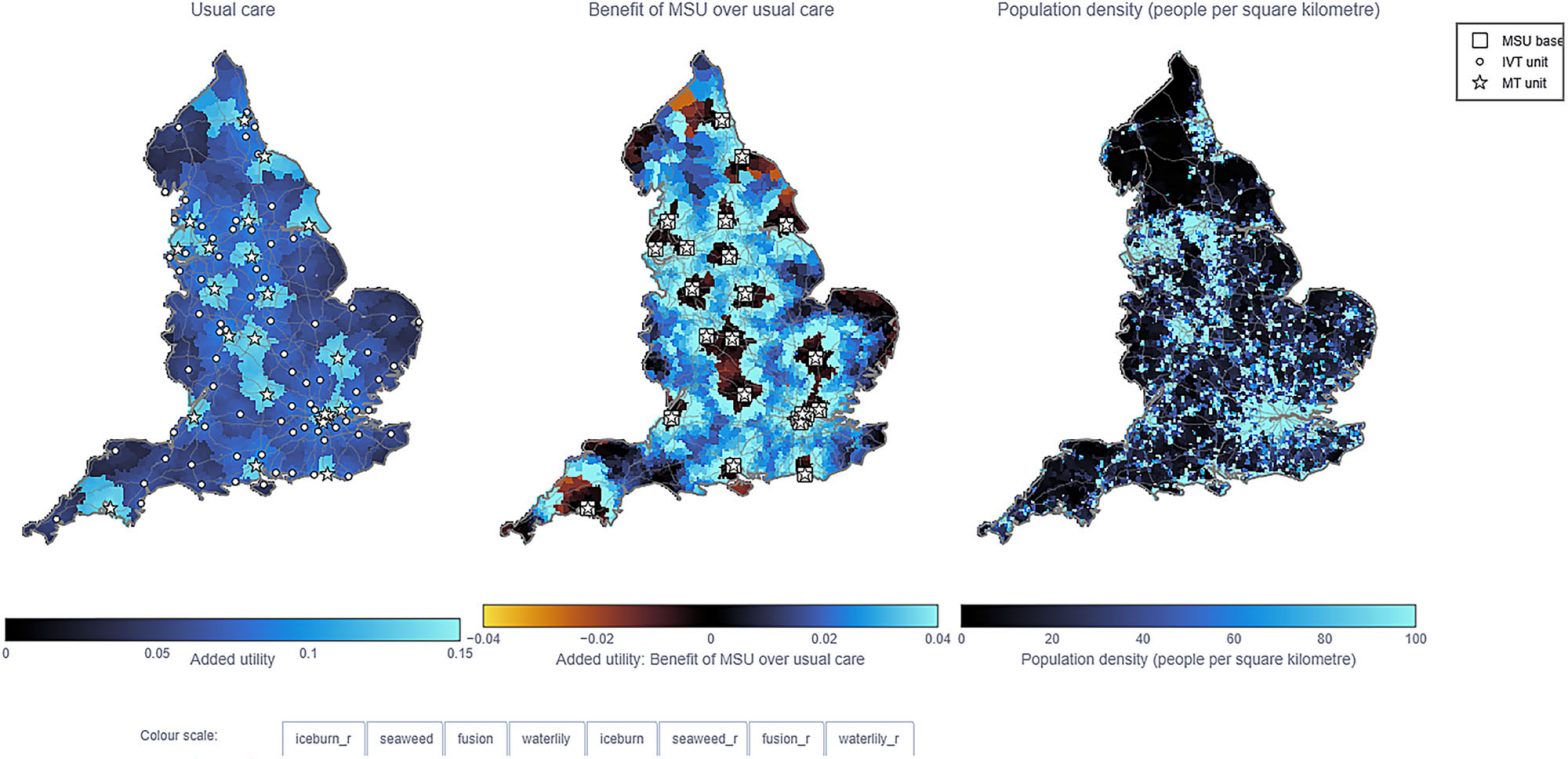
Updated web application maps post-interviews. All maps include major arterial roads, with the left map presenting usual care, the middle map presenting clinical benefit over usual care using added utility, and the right map presenting population density. Population density per lower super output area (LSOA) is calculated by combining data from the Office for National Statistics on population per LSOA in 2018 and on the area of LSOAs in 2011. The default maximum population density of 100 is chosen to show the locations of large towns and city boundaries. All maps include zoom functionality.

Participants felt that as MSUs would likely be managed by the ambulance service, having the locations of ambulance stations available would be beneficial, particularly when zooming into specific localities or regions. Lastly, participants felt that a number of selectable “overlays’’ would be helpful which could show population density and demographics. These were then included in an update to the web application (Fig. 5).

*“…if you could integrate that with population density, that might be helpful… I suppose the older population, people over the age of 50 or 60, adding that as part of your population density map, accepting that stroke is commoner in later life…absolutely [include deprivation]…with deprivation, of course, you’re going to see that, in terms of scale and numbers, more in urban areas than in more rural areas…” (Participant 2, ambulance service staff)*.

### Informing commissioning and implementation decision-making

Usability testing of the web application provided the opportunity to examine commissioning and implementation of MSUs including how the web application could be utilised. As a result of the usability testing we identified that commissioning of MSUs is unlikely to be a straightforward technical, data-informed process. Instead, it would be a complex socio-technical process based on multiple priorities. This is reflected by a participant with experience of working with commissioners, who felt that commissioners would also consider other healthcare conditions.

*“I, honestly, can’t see commissioners using this [web application]. I think it’s great for somebody like me, that’s overseeing huge projects across a regional footprint or, indeed, at system level. So I think it’s great for the providers, it’s great for the network, and you could say it would be great on an urgent and emergency care element as well… As far as our commissioners… certainly, at the moment, there is so little emphasis on stroke anyway that just keeping stroke on the radar at all is tricky” (Participant 5, ambulance service staff)*.

Other participants disagreed as to whether commissioners would use the web application directly to inform commissioning decisions. In the cases where it was thought they would not use the web application it would still support commissioning decisions by being able to provide the evidence for a business case put forward by services or to justify why MSUs should not be commissioned. This contention between participant views reflects the uncertainty that exists in commissioning a new innovation, including knowing who has responsibility for obtaining and examining evidence. This represents the social processes involved in commissioning, where a new innovation would likely then require a local champion.

*“…I think commissioning at a national level would use this and appreciate it. I don’t suspect they get this from many other things that we ask to be commissioned” (Participant 8, stroke or critical care physician)*.

*“…I think that, for an ambulance-based resource, it would probably be for us to make the case [for MSUs], but, you know, share the data, and give the Integrated Care Boards, the commissioning bodies, access to view this to support the conversation so they could see for themselves almost the benefits from it…” (Participant 6, ambulance service staff)*.

Participants also discussed how the wider healthcare economy would contribute to MSU commissioning decisions. For instance, implementation of MSUs may result in some ASU receiving fewer patients and thus make their own services financially unviable due to a loss of the tariff they currently receive for administering IVT. There were also conflicting views amongst participants regarding national or regional commissioning of MSUs. Some participants believed MSUs would need to be part of either a new or existing specialised commissioning pathway, such as the national commissioning of MT, whereas others thought MSUs would be commissioned as part of standard block commissioning contracts with integrated care boards in partnership with local ambulance services. However, if regionally commissioned there were further concerns that ambulance services would have to find capital from within their existing budget to fund MSUs but would not benefit from the gains from implementation of MSUs. These concerns further highlight the social, particularly the political, challenges that were identified as a result of using the web application.

*“You’d get the primary stroke centres…they get a tariff don’t they for thrombolysing somebody in the primary stroke centre [ASU]. So they’d lose that and they’d lose some of their metrics for hyperacute stroke management, because in essence, you’re bypassing them to go straight to the CSC. So I think your primary stroke centres might not like [MSUs being commissioned]” (Participant 9, stroke or critical care physician)*

Alongside the potential for the web application to inform our understanding of commissioning decisions either directly or indirectly, the web application also helped to develop an understanding of how it could be used operationally to support a commissioned MSU in an ambulance service. A critical care consultant and an ambulance service staff member both described how they felt the web application could be used as the basis for informing operational decisions once (and if) MSUs were commissioned. Participants recognised that this would likely require further modifications to the web application, particularly by integrating live ambulance service data on response times and predictive geographic models of stroke incidence.

*“…I suppose the beauty of having big data like this and a very visual representation is, when you pick a particular area…to put the origin of the call on this…and if the [origin of the call is] in the correct colour [showing benefit from MSU attendance]…you go or you don’t go.” (Participant 14, stroke or critical care physician)*

*“…it probably would have…benefit…in terms of looking to plan the shift, plan the day, actually looking where we’ve historically seen higher instances of stroke and those that have subsequently turned out as a confirmed diagnosis. So for predicting around where to position the vehicle on a shift-by-shift basis…”(Participant 6, ambulance service staff)*

In discussions with participants who had lived experience of stroke, there were differing views on whether the web application would be useful, or even be used, by the general public. Of the participants who did feel the web application might be used they explained it was because those people would likely have an interest in stroke care and developing treatments, as described by participant 12 who led a regional group for stroke survivors, *“…Our group would be [interested in the web application]. They’re very open and want to learn more”.* However, it was also highlighted that the data and visual representation from the web application would still be of benefit to the public in educating about, and gaining public support for, MSUs. This potential use was described by a person with lived experience of stroke and a critical care consultant:

*“…maybe if it was London and they were told this won’t- It might actually worsen outcomes, then people might not be for it, or as interested. But…those regions where it would help… I think, yeah, people would definitely be interested.” (Participant 11, patients and public involvement representative)*

*“…if you’re going to commission anything, you have to get the public on board. You have to get people to understand what they’re going to get out of this, not in terms of clinical outcomes, not in terms of value for money, but actually, what does this mean for me tomorrow? And the patient public voice is really important…it’s a key aspect of stakeholder engagement.” (Participant 14, stroke or critical care physician)*

### Informing views on intervention viability

Once participants had used the web application and explored some of the early modelling data presented within it, they then provided their views on whether MSUs would be viable, thus providing an additional use for the web application. In doing so, participants discussed how the maps showed relatively minimal gains to patient outcomes with consideration to how many patients an MSU could realistically see in one day, and how many of those patients would be having an ischaemic stroke and then be eligible for treatment. Alongside this, participants highlighted that the most gains appeared to be for patients with an LVO, where the LVO would be recognised on the scan in the MSU, and patients could be directly transported to a CSC that is able to deliver MT, and thus avoiding secondary transfers. However, one member of the ambulance service explained this may not be an accepted approach by the CSCs. Specifically, they felt it was unclear whether the CSCs would review MSU scans obtained using the onboard CTA scanner and if they would be of sufficient technical quality for a direct admission decision to be made. Whilst the following quote may not accurately represent how CSCs receive MSU patients it demonstrates the uncertainty in how MSUs would integrate with CSC processes:

*“The issue…is…the… [MSU CTA] scanner has got quite a narrow hole, it doesn’t do the neck vessels. So, the MT centres [CSCs] won’t accept those patients [scanned on an MSU] because they would have to repeat the CTA, and then they [might] find they can’t get past the carotid [artery in the neck required to perform MT]” (Participant 4, ambulance service staff)*

Alongside this, participants also highlighted that the maps showed the most gains away from CSCs, typically based in large urban centres, and appeared to make the most improvement in rural areas where travel times to a hospital are the longest. This, again, raised the question of how efficient MSUs would be in these areas, given the relatively low population levels, but participants also recognised that improving access in these areas was important to reduce inequity in access to stroke care. Further, participants discussed the relative levels of poverty in some of these areas, such as some coastal communities, alongside higher age demographics in rural areas, both of which increase the risk of stroke. The difficulty in making this assessment was summed up by a critical care consultant using hypothetical numbers:

*“I don’t know the genuine numbers…but if you look at the tip of Cornwall for instance, so beyond Truro, should we place an MSU down there at the cost of £500,000 that will be used five times a week? Or should we place two MSUs in the middle of Plymouth that will cost £1* *m in total but be used 20 times a week? The former gives you equity of access to the MSU. The latter gives you volume of patients and the number needed to treat for MT” (Participant 14, stroke or critical care physician)*.

Lastly, participants highlighted that MSUs were unlikely to provide the same level of benefit as investing money directly into MT services and widening its availability. However, they recognised that the main issue with increasing MT services was that there were not enough trained neurointerventionists and therefore a like-for-like comparison on benefit was not possible and if expansion of MT services was not immediately available that MSUs could go some way to improving stroke care. This was explained by a stroke physician:

*“…I think if you’re realistic there’s no point commissioning more thrombectomy centres because we can’t staff the ones we’ve got. There’s no point pumping money into thrombectomy centres…We can’t train or recruit the specialists we need to deliver at thrombectomy centres. I don’t think throwing money at thrombectomy centres helps.” (Participant 8, stroke or critical care physician)*

### Trustworthy and robust data

Throughout all of the interviews there were reoccurring discussions about the need for health economics data. Participants felt strongly that incorporating the health economics into the web application would make it more useable in terms of informing their views on the viability of MSUs. They felt this would assist them in understanding not just the potential gains to individual patient outcomes, but also understanding the financial implications, which would make the web application even more useful for informing both commissioning and operational decisions regarding MSU implementation. The need for this was discussed by an ambulance service staff member:

*“I think the health economic bit would be important and an understanding of the numbers of patients that you could expect an MSU to attend in a 24* *h period or a 12* *h period, etc.” (Participant 2, ambulance service staff)*

The inclusion of health economics data was also thought to increase the chances of the web application being used directly by commissioners as this was seen to be a key element of their decision making. Without health economic data, stakeholders involved in commissioning decisions would not have all the information they needed. The emphasis on health economic data was explained by a patient and public involvement representative:

*“That is going to be really powerful, once that’s in [the health economics data], in terms of commissioning” (Participant 17, patient and public involvement representative)*

Finally, the health economics data was highlighted as essential to understanding how commissioning decisions would be informed by debates surrounding how best to improve equitable access to stroke care through implementing MSUs. For example, in terms of rural and urban inequity it was felt that MSUs would need to show they would be economically viable by seeing enough patients, and that this would likely influence any decisions made about the extent to which MSUs could be used to reduce inequities in access to stroke care.

*“The health economic benefit of that, both the number of individuals who can help, but also the utility…Is the balance not that you just have to provide this to the most people you possibly can and accept there are going to be areas you cannot cover well?” (Participant 8, stroke or critical care physician)*

## Discussion

This study presents empirical evidence for how a digital web application can be used to both inform evidence-based commissioning of innovations in the context of acute stroke care and to explore the complexity of commissioning decisions amongst stakeholders in the context of usability testing. Some design elements were identified as requiring changes, the majority of which were specific to the implementation of MSUs in England; highlighting the importance of incorporating qualitative co-design into an agile development process for digital web applications to ensure that they are fit for purpose for all stakeholders involved in commissioning processes.

More specifically, our findings emphasise that digital web applications which convey complicated information require, at minimum, brief training and tooltips to guide the user. The use of co-design in healthcare^30^ and agile processes in application development^35^ are now well established. The benefits of incorporating these approaches is now being recognised with examples of co-production and agile processes being used together in the development of health monitoring applications^36^, assistive technologies^37^ and environmental monitoring^38^. It is acknowledged that building innovative technology is not possible without recognising the context within which they would deployed^39^. Thus co-design, allowing for understanding of the social and professional contexts, alongside agile processes allowing for rapid change of the web application, ensure both its usability and that the developed application is attuned to the complex relational and transactional process that underpin it^26,27,40^. However, this approach also has challenges. There is a requirement for negotiation, at times between the requests put forward by those involved with the co-production and what is achievable in development which requires both parties to have clear discussions and compromise^41,42^. Lastly, it is also important that the process is properly facilitated to be of value to both the co-design participants and the researchers, which requires researchers to buy-in to the process and at times be outside of the comfort zone of traditionally controlled research processes^43^.

The development and usability testing of the digital web application provided an additional benefit in that it allowed us to examine complex issues associated with commissioning that otherwise would not have been possible. We had previously identified several challenges to implementing MSUs in the English and Welsh NHS, including where to locate MSUs, how to staff them, and making dispatch decisions^44^. With a similar group of participants, we have now been able to identify that commissioning is an additional challenge to implementation. Stakeholders using the web application helped highlight the complexities of the commissioning process^24,25^, specifically uncertainty as to who would commission MSUs and whether this would be conducted locally, through centralised commissioning, or linked with specialist commissioning. This coincided with our experiences where, despite informal discussions with various types of commissioners, all declined to participate in the study as they did not feel they would ultimately be involved in the process of commissioning MSUs. The identified uncertainty around responsibility for commissioning is representative of what is known as a “principal-agent problem” resulting from a lack of role clarity. Principal-agent theory is drawn from political and economic science and provides a framework for understanding how work is delegated from principals to agents^45^. In the context of MSUs—where there currently is no policy for their implementation in the NHS—policymakers (principals) would delegate decisions to commissioners (agents), as they do in other complex pre-hospital care commissioning^46^. To address this problem, policy should be developed relating to commissioning decisions for MSUs in the NHS including the identification of who the agents in this instance would be.

The web application also helps address a number of the previously identified barriers identified in the use of research in commissioning decisions, specifically that the web application would not be behind a paywall and was co-designed^25,31^ with a focus on informing commissioning decisions^28^. The data presented within the web application can be used both on a national and local level, with the maps making the data easier to interpret quickly^28,29^. Consequently, the web application would most likely be used by people developing and presenting the case for MSUs to be commissioned rather than commissioners themselves, providing opportunity for the exploration and presentation of context of both existing and alternative services. In doing so, using a web application helps to reduce information access differences (asymmetry) that may exist between commissioners and providers, which is important for optimising commissioning decisions^47^. However, this would only apply where the information is used in support of MSUs as opposed to using the web application to inform when MSUs should not be commissioned, and thus also represents a potential moral hazard that needs to be considered when commissioners review business cases.

The use of the web application also allowed us to further explore some of the previously identified implementation challenges as well as identifying new challenges. One of the keys areas of debate was in relation to the base MSU location and particularly whether MSUs should be based in an urban area, where they might be of benefit to more of the population, or whether the focus should be on rural areas that have poorer access to acute stroke care. This challenge was further discussed following use of the web application which showed higher gains for those further from hospitals, such as rural areas, however participants remained concerned about how much the MSU would then be utilised. As a result of the agile process adopted the web application was able to be updated to include population density which greatly assists in making such a determination and was thought to be essential to support commissioners to balance value-based and cost-effective decisions^20^. The use of the web application also raised the concern about minimal gains to patients, as shown by the utility shift, particularly those without an LVO. This had not been a previous concern, however participants then questioned whether MSUs should targeted towards those with an LVO. Of note in that respect, the literature to date has shown conflicting results about whether or not MSUs speed up access to MT for those with LVO^7,8,14^.

Lastly, the use of the web application highlighted the need for the health economics data to be incorporated into the web application, in line with the need for potential new services to be both of clinical benefit and financially viable^20^. Previous studies have shown that MSUs have the potential to be cost-effective^48–51^ however this was very context dependent, as often seen in economic evaluations^52^. None of these efficacy studies were completed within a UK context, MSUs were trialled in urban areas, and it was reported that MSUs only became cost-effective once they reached a certain patient threshold. Recent work^53^ has shown that MSU cost-effectiveness is extremely contextual in the English NHS, reflecting population density, socio-economic influences on outcomes, the local geography of stroke service providers and quality-adjusted life year thresholds. Therefore, we would not recommend application of a national or generalisable cost-effectiveness calculation into a web application that models national implementation of MSUs, and further sophistication of our model to reflect these important factors is necessary. There still also remains an area of political consideration likely to affect commissioning decisions, which is not addressed in relation to the loss of IVT tariffs for ASUs if MSUs were delivering IVT. This could have an impact on the acceptability of MSUs which is a key element to the successful implementation of new innovations^54,55^. This again highlights the complexities of commissioning and that even when something is evidenced, close relational involvement is still required^26,27^. Taking into consideration the commissioning complexities identified in this study means that setting a decision-making threshold for commissioners, based on NMB or other singular metrics as part of a single technology appraisal, is unlikely to be the single deciding factor in whether MSUs are commissioned. The use of a digital web application modelling the impact of implementing MSUs has been vital to identifying and understanding this challenge.

A strength of the study was that it was conducted using real-world data for the implementation of MSUs in England, ensuring that the models underpinning the usability testing provided accurate representations of MSU implementation, subject to recognised implementation challenges identified elsewhere in the study^44,56^. However, this also contributes to a limitation of the study; the usability testing does not represent how commissioners would actually use the web application as part of the relational decision-making that occurs in commissioning. This requires further evaluation should MSUs be commissioned in the NHS. Whilst some participants might be involved in commissioning processes (we are unable to describe exact job roles to protect participant anonymity), the study would have benefited from further commissioner involvement. However, this was challenging due to the uncertainties identified in the findings as to who would have responsibility for commissioning MSUs. This represents a circular dependency borne from modelling a potential innovation in the NHS that has no current existing commissioning pathway or policy. Other methods would be required to address this circular dependency, such as the use of vignette-based interviews that could present concrete hypothetical situations to commissioners. These vignettes would need to include hypothetical policies to address the previously described principal-agent problem resulting from a lack of role clarity.

In conclusion, a combination of agile processes and co-production in the development of a web application allowed us to develop and present usable models on the effectiveness of MSUs within the English NHS. Usability testing of the web application identified areas for improvement and helped identify complexities in commissioning processes including the lack of role clarity around who would be responsible for the commissioning of MSUs in the absence of clear policy. Further implementation challenges associated with MSUs were also identified, particularly in relation to potential minimal health gains and that maximum gains are seen within rural areas that are unlikely to have the population density required to make an MSU financially viable. This study provides empirical evidence in support of developing innovative and accessible digital dissemination tools to inform commissioning processes. Future research should evaluate the implementation and use of tools to inform commissioning processes, including the relational aspects of commissioning.

## Methods

### Digital web application development

We built models of clinical benefit of MSUs^57^ and aimed to make those models available to potential end-users—including stakeholders involved in commissioning processes—as a digital web application. The chosen method of web application development was to follow an *agile* process^35^ where iterative development, with user feedback, is used rather than a highly developed starting specification. User feedback was incorporated throughout the modelling and web application development via a process of qualitative co-design with stakeholders, recognised to be of importance for helping to ensure clinical relevance^58^. Model code development took place on GitHub (https://github.com/stroke-modelling/muster_workshop_web_app_1) and Streamlit (https://streamlit.io/) was chosen as the deployment platform as this provides a platform designed for modellers and data scientists to easily implement web applications. Initial ranges and default values for the model parameters (which the end-user can change) were reached after examining published information on timings from MSU clinical trials (see Chen et al.^59^ and Fatima et al.^60^ for reviews). The choice of user-controlled input parameters and ranges for their values, and preferred model outputs, were then discussed with stakeholders using nominal group technique^61^ to examine their appropriateness and develop consensus. Some examples of the web application interface are provided in the findings section.

### Study design

The study tested the usability of the developed web application using qualitative think aloud methodology^62^. Think Aloud is a common methodology in usability testing and asks participants to use the web application while talking out loud about what actions they are performing and why, and to provide thoughts on usability. We expanded the usability testing to examine how the web application could inform commissioning decisions in the context of deploying MSUs in England.

### Participants, recruitment and sampling

Participants were recruited via purposive, opportunistic and snowball sampling, as part of the wider research project in relation to MSUs^63^ Stakeholders consisted of stroke clinicians (doctors and nurses), ambulance service staff, decision-makers involved in commissioning processes, and those with lived experience of stroke care (either as a stroke patient or supporting someone impacted by stroke). The research team used existing networks to recruit staff participants and the recruitment of patient and PPI was supported by a national charity, the stroke association. PPI representatives who had expressed an interest to the stroke association in being part of research projects were contacted by the charity with an overview of the study and individuals responded if they wished to take part. The researchers then purposively sampled those who had responded, to ensure diverse representation, based on geographic location, age and type of stroke.

### Data collection

All data were collected online via Microsoft Teams between September and October 2024 using interviews that consisted of a free-form think-aloud component followed by semi-structured discussion. All discussions were audio recorded and transcribed verbatim. Prior to data collection the research team developed a topic guide to cover key aspects of usability based on the major elements of the web application. These were used to facilitate reflection and discussion amongst participants during the think aloud component, particularly during periods of silence and also to guide discussion during the semi-structured component of the interview. Prompts included: what they liked/disliked about the app; ease of use; any additional information they would like to see; how this might be used by commissioning and in wider practice and how the information influenced views on MSUs. Data collection was conducted by JS and LM.

Participants were given a guided tutorial of the web application and the various functions by JS. Participants were then asked to access the web application themselves and share their screen with the researchers while using it. Both researchers observed how users engaged with the web application, whether certain parts of the application were used more than others and areas that participants required additional guidance to utilise. To ensure participants were not digitally excluded from participating they were also given the option for the interviewer to use the web application with the participant telling the interviewer what to do and giving their thoughts.

### Data analysis

Following data collection all interviews were transcribed verbatim and analysed using Nvivo 12 (Lumivero)^64^ by L.M. To consider data from participants’ actions that were seen during the usability rather than verbally recorded L.M. and J.S. met after each interview to compare observations on the participants’ use of the web application. These observations were incorporated into the analysis. Thematic analysis^65^ was used by L.M. to create initial themes exploring commonalities in the data as well as contrasts, with additional input from J.S. The themes were further scrutinised and refined with input from the wider research team resulting in the finalised themes presented.

## Data Availability

The qualitative datasets generated and/or analysed during the current study are not publicly available due to not having ethical approval or participant consent for the sharing of recordings or transcripts beyond select quotations in publications. The corresponding author (J.S.) can interrogate the data on behalf of others upon reasonable request up until 30 April 2032, after which all data will be deleted in line with university data retention policy. Ethical approval will be required for any re-use of data.

## Code availability

The underlying code for this study is available on Github: https://github.com/stroke-modelling/muster_workshop_web_app_1 (version v0.0.11). There are no access restrictions.
